# Multimedia Technology of Spatial Data Mining Based on Genetic Algorithm

**DOI:** 10.1155/2022/4835359

**Published:** 2022-05-21

**Authors:** Yingxin Sun

**Affiliations:** Department of Information Engineering, Changchun College of Electronic Technology, Changchun, Jilin 130000, China

## Abstract

In order to make key decisions more conveniently according to the massive data information obtained, a spatial data mining technology based on a genetic algorithm is proposed, which is combined with the k-means algorithm. The immune principle and adaptive genetic algorithm are introduced to optimize the traditional genetic algorithm, and the K-means, GK, and IGK algorithms are compared and analyzed. The results show that, in two different datasets, the objective functions obtained by the K-means algorithm are 94.05822 and 4.10373 (×10^6^), respectively, while the objective functions obtained by the GK and IGK algorithms are 89.8619 and 3.9088 (×10^6^), respectively. The difference between the three algorithms can also be reflected in the data comparison of the number of iterations. The number of iterations required for k-means to reach the optimal solution is 8.21 and 8.4, respectively, which is the most among the three algorithms, while the number of iterations required for IGK to reach the optimal solution is 5.84 and 4.9, respectively, which is the least. Although the time required for K-means is short, by comparison, the IGK algorithm we use can get the optimal solution in relatively less time.

## 1. Introduction

**Figure 1:**
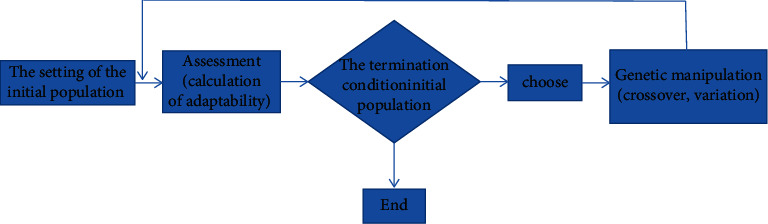
Basic steps of genetic algorithm.

Spatial data mining is data mining and knowledge discovery in a spatial database. It belongs to a branch of data mining. It mainly obtains some spatial features and patterns that users are interested in, the relationship between spatial data and nonspatial data, and the characteristics of universal data hidden in the database. However, there are many differences between spatial data mining and conventional data mining, which are also different from general affairs' data mining. Spatial data mining expands many spatial scale dimensions in the spatial theory of state discovery [1]. Because spatial data itself has complex and diverse characteristics, its mining technology is very different from the conventional thing data mining technology. The main characteristics of this technology are as follows: spatial data mining technology has rich data sources, huge amount of data, complex access methods, and various data types; spatial data mining has a wide range of application fields, which are closely related to all the data about spatial location; the algorithms and methods of spatial data mining technology are unusual, and their algorithms are usually difficult and complex; there are many ways to express the knowledge of spatial data mining technology, which mainly depends on people's understanding and cognition of the technology. Genetic algorithm (GA) is a computational model that simulates the biological evolution process of Darwin's genetic selection and natural elimination. It was first proposed by Professor Holland J. of Michigan University in 1975. In the sense of simulating the evolution process, GA is a slow-moving structure, which can adopt a variety of different composition schemes. This is mainly reflected in parameter coding and genetic operation technology, which are related [2]. At present, the main coding techniques of the genetic algorithm include one-dimensional chromosome coding, multiparameter mapping coding, discretization, variable chromosome length coding, and two-dimensional chromosome coding. Genetic manipulation technology is embodied in three aspects: selection probability, crossover probability, and mutation probability [3]. The basic steps of the genetic algorithm are shown in Figure 1.

## 2. Literature Review

**Figure 2:**
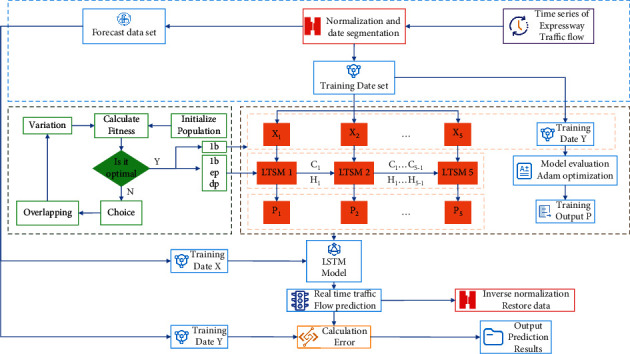
Flowchart of data mining technology.

Data mining is to analyze the observed datasets (often huge), in order to find unknown relationships and summarize data in a novel way that data owners can understand. The value of data mining is shown in Figure 2. Saha et al. said that, from a technical point of view, data mining is a process of extracting hidden, unknown but potentially useful information and knowledge from a large number of incomplete, noisy, fuzzy, and random actual data [4]. Fallahpour et al. said that, from the perspective of business, data mining is a new business information processing technology. Its main feature is to extract, transform, analyze, and model a large number of business data from the business database so as to extract the key knowledge to assist business decision-making, that is, to find relevant business models from a database [5]. The research of Zhang et al. shows that the emergence of large-scale database, advanced computer technology, the actual needs of operation and management, and the deep computing ability of these data promote the birth, rapid development, and wide application of data mining [6]. Renić et al. said that data mining is actually the result of the gradual evolution of information technology and the result of people's long-term research and development of database technology [7]. Mahbuby et al. found that, with the development and maturity of three basic technologies, massive data collection, powerful multiprocessor computer, and data mining algorithm, data mining technology began to receive extensive attention in commercial applications [8]. Balasubramanian and Rajendran said that genetic algorithm is the most important technology among many mining technologies. It can filter information from a large amount of data in the data warehouse, find possible operation modes in the market, and mine facts that people do not know [9]. Xu et al. said that databases generally have thousands of tables and attributes and millions of tuples. The gigabit level data in the database are no longer rare because the trillion level number database has been born, replacing the gigabit level database [10]. Liu et al. obtained through research that the massive database in high-dimensional space not only makes the search space larger but also makes it easier to find pattern errors. Therefore, make full use of relevant knowledge to change the dimension, reduce the dimension, and delete redundant data so as to make the data mining algorithm more efficient [11]. Oliva et al. said that the algorithm for providing knowledge from massive spatial data should be testable and efficient [12]. Zhang et al. proposed that polynomial and exponential algorithms have no practical value, but if the algorithm is changed into a specific model with limited data to obtain appropriate parameters, the value will be considerable [13].

## 3. Methods

### 3.1. Coding Scheme and Population Initialization

Firstly, how to encode is the first step in the evolutionary design of a genetic algorithm. For the clustering analysis of the K-means algorithm, the amount of data is large and multidimensional. If binary coding is adopted, the search space of data will increase sharply, which will greatly reduce the efficiency of calculation. Therefore, this study adopts real coding [14]. The initialization of the K-means algorithm is the initialization of the cluster center, and we should set the appropriate cluster center. First, we encode the cluster center. Now, assuming that the coordinates of the cluster center are *d*-dimensional, for K clusters, the length of each chromosome is *k∗d* and the chromosome is {*p*_1_, *p*_2_, *p*_3_,…, *p*_*k*_}, where *p*_*i*_=⌊*p*_*j*1_, *p*_*j*2_,…, *p*_*j*  *d*_⌋ randomly selects *K* from *n* objects as the center coordinates of the initial cluster for each corresponding chromosome. We are now going to cluster huge data. Theoretically, if the number of initial populations is large, there will be many problems. However, in order to diversify the data elements, we should set the population number as large as possible under the conditions allowed by various facilities [15]. In fact, the specific initialization process is to select *k* of the *N*-target classification objects as a solution to the problem and encode them into a chromosome. Repeat this for *m* times, select *m* chromosomes, obtain the initial population, and complete the population initialization [16]. The ultimate goal of clustering is that, in the obtained clustering, the smaller the distance between similar objects, the better the clustering effect. On the contrary, the greater the distance between different objects, the better the clustering effect. According to such requirements, we select the following fitness function:

(1) $$\begin{array}{c} f=\frac{1}{E}=\frac{1}{\sum_{i=1}^{k}\sum_{p\in c_{i}}{\left|p-p_{i}\right|}^{2}}. \end{array}$$

From the above formula, we can see that *f* is nonnegative, and when *f* increases, the criterion function *E* decreases, and the clustering effect is obviously improved. On the contrary, when *f* decreases, the criterion function *E* increases, and the clustering effect is obviously poor. The so-called fitness function can well reflect the quality of clustering results, so as to screen the optimal solution. After determining the fitness function, considering that, in the early stages of evolution, if the offspring of excellent individuals will account for a large proportion of the whole population, the fitness values will be relatively close in the later stages of evolution. At this time, the advantages of the offspring of excellent individuals are not highlighted, and the evolution of the whole population is almost stopped when making selection operations [17]. In view of the problems of premature convergence (premature) in the early stage and slow convergence in the late stage, we also need to process it. Now, we combine genetic algorithm with simulated annealing algorithm and stretch the fitness appropriately with simulated annealing algorithm so that the dominant individuals can be in a very obvious position in the whole evolution process, so as to ensure the smooth progress of the evolution process. The specific stretching method can be seen as follows:

(2) $$\begin{array}{c} f_{i}=\frac{e^{f_{i}/T}}{\sum_{i=1}^{M}e^{f_{i}/T}},\\ T=T_{0}\left(0.99^{g-1}\right), \end{array}$$

where *f*_*i*_ is the fitness of the *i*th individual, *M* is the population size, *g* is genetic algebra, *T* is the temperature, and *T*_0_ is the initial temperature.

### 3.2. Selection Operation

In order to better carry out the selection operation and overcome the premature problem of genetic algorithm, we adopt a selection operation based on the immune principle to adjust the selection probability [18]. The immune optimization algorithm draws lessons from the evolutionary mechanism of the biological immune system and combines the idea of traditional genetic algorithm, which can accurately improve the performance of the algorithm to a certain extent. When introducing an immune mechanism to solve the problem, we can first take the objective function and constraint conditions as the antigen input, then generate the initial antibody population, then calculate a series of genetic operations and antibody affinity, and finally find the antibody against the antigen while maintaining the antibody diversity:

(3) $$\begin{array}{c} d=\frac{N_{m}}{p_{\text{size}}}, \end{array}$$

where *N*_*m*_ is the number of identical individuals in the population and *p*_size_ is the size of the population. Find out the *m* individuals with the largest individual concentration in the population, set as 1,2,…, *m*; then, the individual concentration probability of these *m* individuals:

(4) $$\begin{array}{c} p_{d}=\frac{1-d}{p_{\text{size}}}. \end{array}$$

The concentration probabilities of the remaining individuals are P, and the sum of the concentration probabilities of all individuals is 1. If the fitness of an individual is *f*_*i*_ and the probability of the individual being selected is *p*_*f*_*i*__, then

(5) $$\begin{array}{c} p=\frac{f_{i}}{p_{d}\times \sum_{j=1}^{p_{\text{size}}}f_{i}}, \end{array}$$

where *i*=1,2,3,…, *p*_size_; through the above definition of individual concentration, it is more convenient to select the best individual and improve the accuracy of selection. It can be seen that the greater the fitness of the individual, the greater the probability of being selected, which accelerates the convergence of the algorithm. It can also be seen that the greater the individual concentration, the smaller the probability of being selected, so as to ensure the diversity of all individuals in the evolutionary population and prevent premature convergence. The parameter crossover probability *p*_*c*_ and mutation probability *p*_*m*_ of genetic algorithm are very important to the effect of genetic algorithm, which will directly affect the convergence of the algorithm. Too large or too small crossover probability *p*_*c*_ and mutation probability *p*_*m*_ will greatly affect the performance of the algorithm. Therefore, it requires repeated experiments to determine the crossover probability *p*_*c*_ and mutation probability *p*_*m*_ for different optimization problems, so as to find the best crossover probability *p*_*c*_ and mutation probability *p*_*m*_ suitable for specific problems [19]. Kalliamvakou et al. proposed an adaptive genetic algorithm. Now, we can use it to solve the above problems [20]. In the adaptive algorithm, and it can be adjusted dynamically with the fitness. If the average fitness value of the population is lower than the fitness value, the individual corresponds to a lower crossover probability *p*_*c*_ and mutation probability *p*_*m*_, and the individual is protected and enters the next generation. On the contrary, if the average fitness value of the population is higher than the fitness value of the individual, the individual corresponds to a higher crossover probability *p*_*c*_ and mutation probability *p*_*m*_, and the individual will be eliminated. Therefore, the crossover probability *p*_*c*_ and mutation probability *p*_*m*_ adjusted by the adaptive algorithm provide the best crossover probability *p*_*c*_ and mutation probability *p*_*m*_ relative to a solution, which not only maintains the population diversity but also ensures the convergence of the genetic algorithm [21].

### 3.3. Multimedia Technology

The meaning of multimedia is to combine the TV type audio-visual information dissemination ability with the computer's interactive control function. Creating a new information processing model integrating text, pictures, sound, and image, making the computer have the functions of digital, fully dynamic, and full video broadcasting, editing and creating multimedia information, and controlling and transmitting multimedia e-mail, video conference, and other video transmission functions will make the standardization and practicability of the computer a major topic of this new technological revolution. The use and high-speed transmission of digital audio and video data have become a symbol of a country's technical level and economic strength [22]. The world is large, but it is also small, which is true. Multimedia technology can enable people to understand astronomy and geography, history and culture, high and new technology, and local customs across time and space. From the national government to the ordinary people, they are dealing with multimedia technology every day. People's living standards have improved, which can also be seen from the development of multimedia. Communication, digital audio-visual technology, network television, 3G, MP4, MP5, etc., all can reflect the progress of human civilization [23].

## 4. Results and Analysis

**Figure 3:**
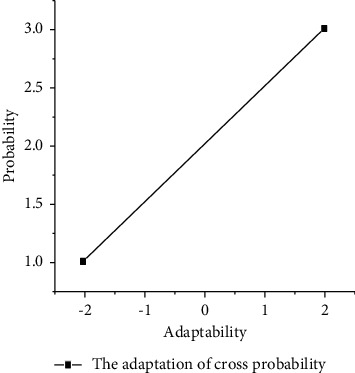
Adaptive of crossover probability.

**Figure 4:**
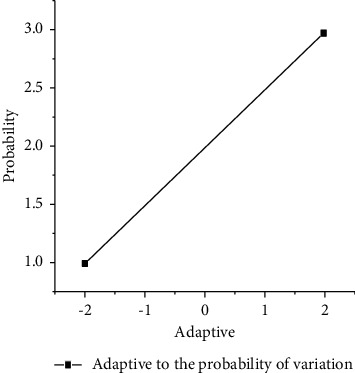
Adaptive of mutation probability.

The crossover probability *p*_*c*_ and mutation probability *p*_*m*_ are adaptively transformed according to the following formula:

(6) $$\begin{array}{c} \left\{\begin{array}{cc} \frac{k_{1}\left(f_{\max}-f{}^{\prime }\right)}{f_{\max}-f_{\text{avg}}}, & f\geq f_{\text{avg}},\\ k_{2}, & f<f_{\text{avg}}, \end{array}\right.\\ \left\{\begin{array}{cc} \frac{k_{3}\left(f_{\max}-f{}^{\prime }\right)}{f_{\max}-f_{\text{avg}}}, & f\geq f_{\text{avg}},\\ k_{4}, & f<f_{\text{avg}}, \end{array}\right. \end{array}$$

where *f*_max_ is the maximum fitness value of the population, *f*_avg_ is the average fitness value of each generation of the population, *f*_min_ is the minimum fitness value of the population, *e* is the larger fitness value of the two individuals to be crossed, and *f*′ is the fitness value of the individuals to be mutated. *k*_1_, *k*_2_, *k*_3_, *k*_4_ take the value of (0,1) interval. If there is no clear basis for the definition of *k*_1_, *k*_2_, *k*_3_, *k*_4_, we can set the initialization value, analyze the crossover probability *p*_*c*_ and mutation probability *p*_*m*_, and compare the evolutionary algebra under the same conditions. The crossover probability *p*_*c*_ and the mutation probability *p*_*m*_ with fewer evolutionary algebras are better [24]. Similarly, in order to better overcome the premature problem, the original adaptive formula is now improved, and the crossover probability *p*_*c*_ and mutation probability *p*_*m*_ are adjusted according to the following formula:

(7) $$\begin{array}{c} p_{c}=\left\{\begin{array}{cc} \frac{p_{c1}\left(f_{\text{avg}}-f{}^{\prime }\right)+p_{c2}\left(f{}^{\prime }-f_{\min}\right)}{f_{\text{avg}}-f_{\min}}, & \left(f\prime <f_{\text{avg}}\right),\\ \frac{p_{c2}\left(f_{\text{avg}}-f{}^{\prime }\right)+p_{c3}\left(f{}^{\prime }-f_{\text{avg}}\right)}{f_{\max}-f_{\text{avg}}}, & \left(f\prime \geq f_{\text{avg}}\right), \end{array}\right.\\ p_{c}=\left\{\begin{array}{cc} \frac{p_{m1}\left(f_{\text{avg}}-f\right)+p_{c2}\left(f-f_{\min}\right)}{f_{\text{avg}}-f_{\min}}, & \left(f<f_{\text{avg}}\right),\\ \frac{p_{m2}\left(f_{\text{avg}}-f\right)+p_{c3}\left(f-f_{\text{avg}}\right)}{f_{\max}-f_{\text{avg}}}, & \left(f\geq f_{\text{avg}}\right), \end{array}\right. \end{array}$$

where parameter *f*_max_, *f*_avg_, *f*_min_, *f*′, and*f* are the same as the previous one. The value range of *p*_*c*1_ > *p*_*c*2_ > *p*_*c*3_, *p*_*m*1_ > *p*_*m*2_ > *p*_*m*3_ (0,1) can be dynamically adjusted during evolution. The specific meaning is shown in the figures, which reflects the change of crossover probability *p*_*c*_ and mutation probability *p*_*m*_ with fitness shown in Figures 3 and 4 [25].

**Figure 5:**
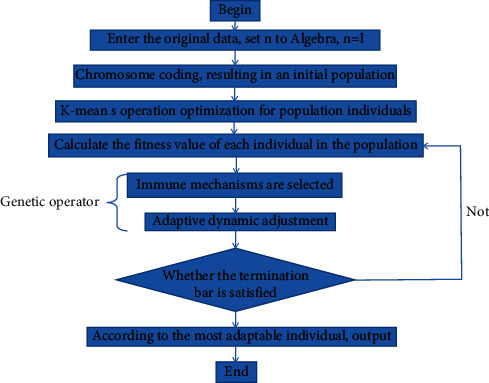
Flowchart.

According to the basic idea of the algorithm described above, the basic flow of the algorithm is shown in Figure 5.

It is mainly divided into three algorithms to test, which are the classical k-means algorithm, the general genetic optimization k-means algorithm (GK), and the improved genetic k-means algorithm (IGK). The test environment is programmed and tested with MATLAB on an ordinary PC. The experimental data adopts two classic datasets of machine learning (UCI Machine Learning Repository), Iris and Glass. The above datasets are internationally recognized typical datasets for testing the performance of clustering methods [26]. The Iris dataset has 150 sample information, each sample information includes 4 attributes, a total of 3 categories, and each category has 50 samples. There are 214 sample information in the Glass dataset, and each sample information includes 9 attributes, a total of 6 categories.

**Figure 6:**
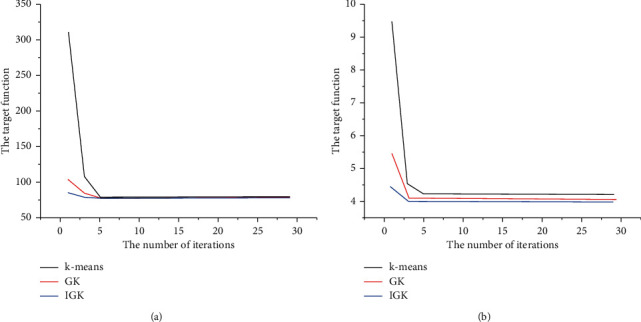
The variation curve of the objective function value of the three clustering algorithms in the K-means clustering stage with the number of iterations in the clustering experiment of two experimental datasets. The objective function value on the curve represents the average result of 20 experiments. (a) The experimental results of the Iris dataset test show that although the three algorithms finally converge to the value of the same objective function, it is obvious that GK and IGK converge faster than the k-means algorithm, and the IGK algorithm is faster than the GK and K-means algorithm. (b) The experimental results of the Glass dataset test; similarly, it can be seen from the graph that the final convergence values of the three algorithms are very similar, but the average objective function value of the k-means algorithm is actually much larger than the GK amount IGK.

Some parameters of the test experiment are set as follows: the number of clusters *k* = 3, the number of iterations *t* = 100, and the initial temperature *T*_0_ = 10. Population size *M* = 30, the individual crossover probability corresponding to the minimum fitness value *p*_*c*1_ = 0.7, the individual crossover probability corresponding to the average fitness value *p*_*c*2_ = 0.85, the individual crossover probability corresponding to the maximum fitness value *p*_*c*3_ = 0.95, the individual variation probability corresponding to the minimum fitness value *p*_*m*1_ = 0.05, the individual variation probability corresponding to the average fitness value *p*_*m*2_ = 0.1, and the individual variation probability corresponding to the maximum fitness value *p*_*m*3_ = 0.15. The traditional K-means algorithm, the traditional genetic algorithm's k-means algorithm (GK), and the improved algorithm (IGK) in this study are used to test the two datasets, respectively. All the algorithms run 20 times, respectively, and then compare and analyze the performance of the algorithm from the aspects of criterion function value, iteration times, and running time. The specific experimental results are shown in Figure 6. For the Iris dataset, it can be seen that these three algorithms can finally get the optimal solution at 89.8619, but in the 20 times of operation, the k-means algorithm reaches the optimal solution only ten times, and its objective function falls into the local minimum for other times, while the other two algorithms can reach the global optimal solution every time in the 20 times of operation. For the Glass dataset, it can be seen that these three algorithms can also achieve the optimal solution, but the clustering objective function falls into the local minimum four times in the 20 times of the K-means algorithm. The other operations can converge to the optimal solution 3.9088 × 10^6^ every time. Similarly, the other two algorithms can reach the optimal solution every time [27].

It can be seen that the unit in the graph is millions [28]. Therefore, we can also conclude that GK and IGK converge faster than the k-means algorithm, and the IGK algorithm is faster than GK and k-means. From the above analysis, we can see that the convergence speed of the IGK algorithm in the K-means clustering stage is the fastest among the three algorithms. Therefore, the improved genetic algorithm is more suitable for the k-means clustering problem. Let us synthesize the experimental results and compare the performance of the three algorithms from three aspects: objective function value, iteration times, and running time.  Table 1 shows the average results of 20 clustering experiments of the three algorithms on two experimental datasets.

## 5. Conclusion

Firstly, we look at the objective function. In the two groups of experiments, the Iris dataset and the Glass dataset, the objective functions obtained by the K-means algorithm are 94.05822 and 4.10373 (×10^6^), respectively, which are slightly different from the optimal solution. The objective functions obtained by the other two algorithms are 89.8619 and 3.9088 (×10^6^), which are very accurate and reach the optimal solution. In the Glass dataset, the gap is more obvious and smaller than the optimal solution because K-means is easy to fall into a local minimum. Looking at the number of iterations, in the Iris dataset or Glass dataset, the number of iterations required for the k-means to reach the optimal solution is 8.21 and 8.4, respectively, followed by GK algorithm, which is 6.1 and 6.58, respectively, The least number of iterations is our algorithm (IGK), which is 5.84 and 4.9, respectively. Therefore, the genetically optimized K-means can not only ensure the optimal solution but also make the K-means converge quickly. Finally, comparing the running times of each algorithm, the K-means algorithm takes the least time because GK and IGK spend a lot of time in the process of finding the initial cluster center, but compared with this algorithm (IGK), the time required is relatively small under the condition of ensuring the global optimal solution. Aiming at the classical algorithm of clustering analysis technology of data mining, the K-means algorithm is analyzed in detail, and it is improved by combining it with the genetic algorithm. At the same time, it improves the defects of traditional genetic algorithms. Combined with the idea of simulated annealing algorithm, immune mechanism, and adaptive algorithm, the premature problem of genetic algorithm is well optimized to a certain extent. Finally, the improved algorithm (IGK), the standard k-means algorithm, and the traditional genetic algorithm (GK) are compared. Through the analysis of the experimental results, it is proved that the improved algorithm has better clustering effect and the performance is also the best. Research and introduce more methods to improve genetic algorithms, such as the gradient method, the hill-climbing method, or the heuristic algorithm with problem-related heuristic knowledge. If the ideas of these optimization methods are integrated, a hybrid genetic algorithm with stronger performance will be formed. It is also a means to improve the efficiency and accuracy of the genetic algorithm.

## Figures and Tables

**Table 1:** Comparison of average clustering results of various algorithms.

Serial number	Iris dataset			Glass dataset		
	Objective function	Number of iterations	Running time (s)	Objective function (×10^6^)	Number of iterations	Running time (s)
k-means	94.05822	8.21	0.050476	4.10373	8.4	0.065802
GK	89.8619	6.1	7.417521	3.9088	6.58	6.958297
IGK	89.8619	5.84	3.154764	3.9088	4.9	2.862174

## Data Availability

The data used to support the findings of this study are available from the corresponding author upon request.
